# Rapid clinical recovery of a SARS-CoV-2 infected common variable immunodeficiency patient following the infusion of COVID-19 convalescent plasma

**DOI:** 10.1186/s13223-021-00518-5

**Published:** 2021-02-05

**Authors:** Luciana C. Ribeiro, Bruno Deltreggia Benites, Raisa G. Ulaf, Thyago A. Nunes, Carolina Costa-Lima, Marcelo Addas-Carvalho, José Luiz Proenca-Modena, Fabiana Granja, Vitor Antonio da Costa, Adriana da Silva Santos Duarte, Audrey Basso Zangirolami, Emerson Clayton Amaro, Eli Mansour, Ricardo L. Zollner, Licio A. Velloso

**Affiliations:** 1 Department of Internal Medicine, School of Medical Sciences, University of Campinas, Campinas, SP 13083-887 Brazil; 2 Hematology and Transfusion Medicine Center, University of Campinas, Campinas, Brazil; 3 Laboratory of Emerging Viruses (LEVE), Department of Genetics, Evolution, Microbiology and Immunology, Institute of Biology, University of Campinas, Campinas, Brazil; 4 Experimental Medicine Research Cluster (EMRC), University of Campinas, Campinas, Brazil; 5 Biodiversity Research Center, Federal University of Roraima, Roraima, Brazil

**Keywords:** Hypogammaglobulinemia, B-lymphocyte, Infection, Pneumonia, Lung, Computed tomography

## Abstract

**Background:**

Common variable immunodeficiency is the most prevalent symptomatic primary immunodeficiency in adults. Affected patients fail to mount an appropriate humoral response against community acquired infectious diseases and recent reports have provided data supporting the increased susceptibility of these patients to severe SARS-CoV-2 infections. In this context, the infusion of COVID-19 convalescent plasma could represent an effective therapeutic strategy.

**Case presentation:**

25-year old woman diagnosed with common variable immunodeficiency in 2013, developed severe COVID-19 that rapidly progressed to pneumonia presenting with multiple bilateral lung opacities that were both central and peripheral and presented as ground-glass and consolidation types involving all lobes, bilaterally. As blood oxygen saturation decayed and lung abnormalities were not responsive to large spectrum antibiotics and corticosteroids, patient was placed on mechanical ventilation and compassionate-use of approved COVID-19 convalescent donor plasma was introduced. The patient presented a rapid response to the approach and mechanical ventilation could be interrupted 24 h after first dose of COVID-19 convalescent donor plasma. As a whole, the patient received four doses of 200 mL convalescent plasma during a period of 6 days. There was rapid improvement of clinical status, with interruption of supplemental oxygen therapy after 6 days and reduction of lung abnormalities as evidence by sequential computed tomography scans.

**Conclusions:**

This is a single patient report that adds to other few reports on common variable immunodeficiency and agammaglobulinemia, suggesting that COVID-19 convalescent donor plasma could be a valuable therapeutic approach to treat patients affected by dysgammaglobulinemias and presenting severe COVID-19.

## Background

Common variable immunodeficiency (CVID) is the most prevalent symptomatic primary immunodeficiency affecting adults [1–3]. It is characterized by hypogammaglobulinemia, failure to produce specific antibodies and susceptibility to infections [1–3]. Affected patients present poor response to vaccines and defective capacity to mount an adaptive response to pathogens, including viruses [4]. As a rule, CVID patients are treated with immunoglobulin replacement, which promotes efficient protection against most community acquired infectious diseases [2]. The effectiveness of immunoglobulin replacement relies on the fact that commercially available human immunoglobulins are produced from pools of donors, warranting a wide spectrum of antibody diversity. Unfortunately, the recent emergence of COVID-19 could represent a particular risk for CVID patients because currently available immunoglobulins were produced from blood samples donated before, or in the early stages of COVID-19 pandemics, implying that most donors might not have been immunized against SARS-CoV-2. In addition, a recent study has reported that human adaptive memory to coronaviruses is short-lasting, which may suggest that even in the future, the presence of immunoneutralizing antibodies against SARS-CoV-2 in donor immunoglobulin could be scarce [5]. Recent reports have provided insights and data to support the increased susceptibility of CVID and also agammaglobulinemia patients to severe SARS-CoV-2 infections [6–9]. In this context, it has been proposed that infusion of COVID-19 convalescent plasma could represent an effective approach to treat CVID patients developing COVID-19 [10].

## Case presentation

**Fig. 1:**
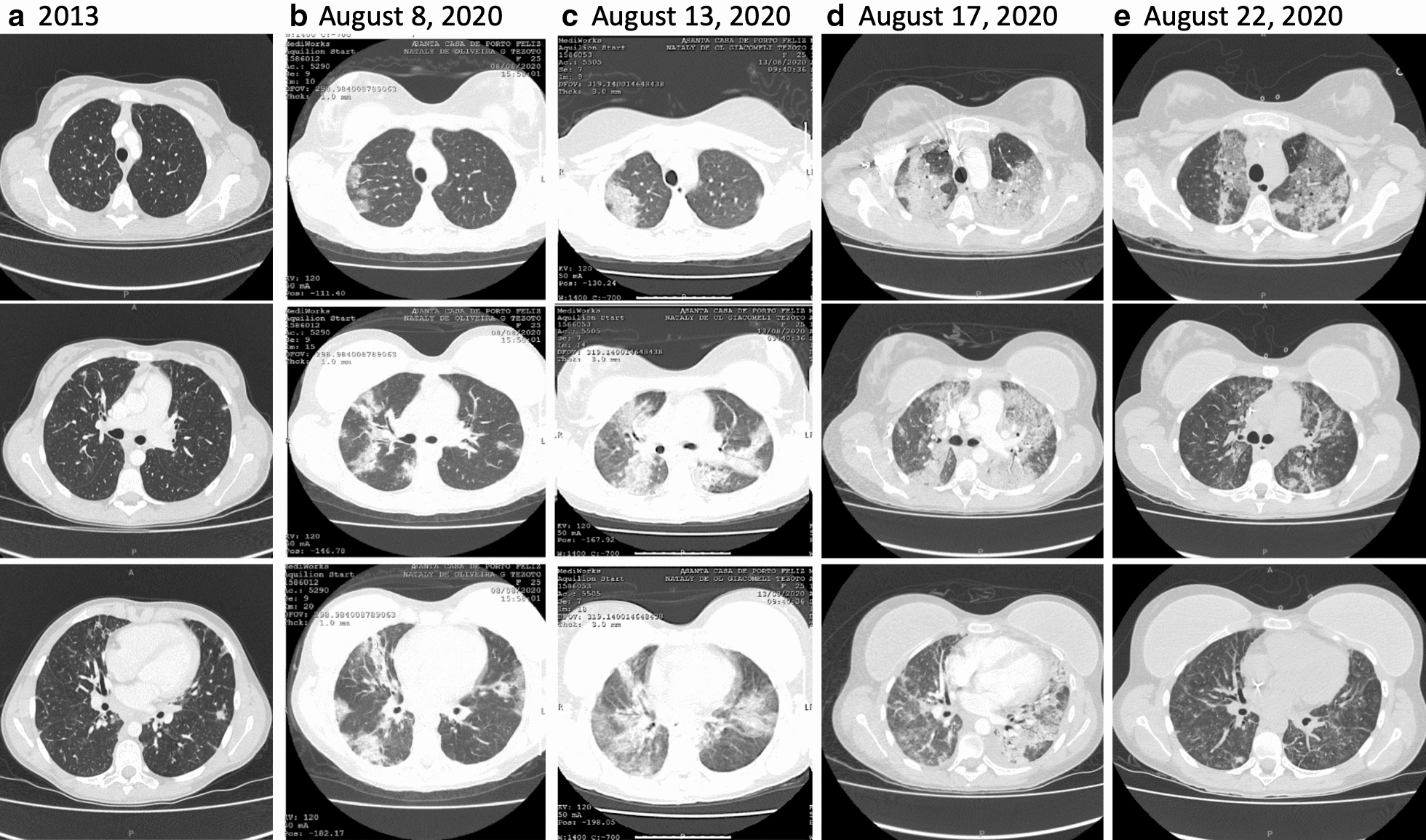
Lung computed tomography scans obtained at the time of common variable immunodeficiency diagnosis, in 2013 (**a**) and during COVID-19, (**b**–**e**). The dates when scans were performed are depicted in the Figure. As for the interpretation of scans, the patient was submitted to a breast implant in 2017

Here, we report the case of a 25-year old woman diagnosed with CVID in 2013 that was infected with SARS-CoV-2 developing severe COVID-19 and presenting rapid response to COVID-19 convalescent plasma infusion. Except for CVID, the patient had no other comorbidity, had no family records for any primary immunodeficiency, was lean and practiced sports frequently. At the time of CVID diagnosis that was defined on the basis of the recommendations of the International Consensus Document (ICON): Common Variable Immunodeficiency Disorders [3], patient reported recurrent infections of the upper respiratory tract and lungs, beginning in 2000 (at the age of 5-y); blood IgG was 0.071 g/L (normal range 5.4–16.1 g/L); IgA was 0.22 g/L (normal range 0.80–2.80 g/L); and, peripheral blood cell immunophenotyping determined 86% CD3, 52% CD4, 31% CD8, 4.1% CD19 and 6.2% CD16 + CD56. In addition, she presented absent baseline and post-vaccinal humoral response to pathogen-derived peptide and polysaccharide antigens. Immunoglobulin replacement was introduced in 2013 and administered regularly without interruptions thenceforth. The initial dose was 400 mg/kg every 28 days and after adjustments based on the stabilization of blood IgG at minimum 8.0 g/L, maintenance dose was defined as 25 g every 28 days (345 mg/kg/dose). From 2013 to 2020, patient was constantly monitored in regular visits to the University of Campinas Clinics Hospital. She was regularly submitted to blood IgG determinations and clinically evaluated for signs of uncontrolled disease, such as infections, autoimmunity, inflammatory conditions and neoplasia. During the entire period, treatment was efficient to keep her on optimal medical condition. The determinations of blood IgG in the months prior to SARS-CoV-2 infection confirmed the effectiveness of the treatment (IgG = 10.04 g/L in November 2019; IgG = 10.01 g/L in July 2020). Lung CT scan at CVID diagnosis (2013) revealed sparse areas of fibrosis and bronchiectasis predominantly in the anterior and superior segments of the right lung inferior lobe; inferior and superior lingular segments of left lung superior lobe and anterior portions of the left lung inferior lobe (Fig. 1a). During follow up, from 2013 to 2020, no additional lung CT scans were performed because patient presented an optimal response to therapeutic intervention.

On August 8, 2020, the patient was admitted at her hometown Hospital with fever (39.8 °C), cough, dyspnea and 95% blood O_2_ saturation. Lung CT scan (Fig. 1b) revealed multiple bilateral lung opacities that were both central and peripheral and presented as ground-glass and consolidation types involving all lobes, bilaterally, suggesting COVID-19 and scoring 9 according to the COVID-19 lung CT scoring system published elsewhere [11]. Despite clinical and radiological evidence for COVID-19, the real-time PCR from a nasopharynx sample was negative for SARS-CoV-2. The patient was administered low-flow supplemental oxygen and treated with antibiotics and corticosteroid. As fever continued, lung CT scan score progressed to 14 (Fig. 1c) and dyspnea progressively worsened, supplemental oxygen was increased to high-flow and on August 13, the patient was transferred to the high-complexity University of Campinas Clinics Hospital. On admittance, a new nasopharyngeal swab was collected, which scored positive for SARS-CoV-2 on real-time PCR. At this stage, blood O_2_ saturation was 91% on ambient air and patient was maintained on high-flux supplemental oxygen. A wider spectrum antibiotic was introduced, and dexamethasone was administered in a dose previously shown to be most effective for severe COVID-19 [12]. Blood O_2_ saturation was continuously monitored and supplemental O2 was adjusted accordingly. Despite intense clinical and pharmacological therapy, fever and dyspnea persisted and on August 17, blood O_2_ saturation was 85% on high-flux supplemental oxygen. A new lung CT scan revealed considerable worsening of the lung damage, scoring 22 (Fig. 1d). The patient was transferred to the intensive care unit and placed on invasive mechanical ventilation. At this stage, total blood IgG was 6.08 g/L, and both sputum and blood cultures were negative for bacterial and fungal infections. On August 19, as no clinical improvement was obtained, we decided for compassionate-use approved COVID-19 convalescent donor plasma (200 mL, 1/1280 SARS-Cov-2 specific neutralizing antibody (nAb) plus 200 mL, 1/320 nAb). The patient’s blood levels of anti-SARS-CoV-2 IgM and IgG antibodies scored negative using two distinct tests (OnSite Beijing Genesee Biotech, Inc., China and Architect Abbott, Ireland) [13].

Each convalescent plasma unit was obtained from a different donor by apheresis using the Amicus™ automated blood cell separator (Fresenius Kabi AG, Bad Homburg, Germany). Donation criteria included: laboratory confirmation of previous SARS-Cov-2 infection through positive RT-PCR test result, absence of symptoms for at least 28 days and eligibility for other clinical and laboratory blood donation criteria in accordance with national legislation. The evaluation of SARS-Cov-2 specific nAb titers was performed by observing the cytopathic effect in cultures of Vero cells incubated with a serum-virus mixture. After 3 days of incubation, Vero cells were inspected by an inverted optical microscope; the highest serum dilution that protected more than 80% of cells from cytopathic effect was taken as the neutralization titer. These procedures were carried out within a clinical trial approved by the Brazilian Commission on Ethics on Research (CONEP, Approval number 4.021.484).

On August 20, the patient presented improvement of respiratory condition with blood O_2_ saturation 100% on mechanical ventilation and placed on progressive reduction of FiO_2_. As fever ceased and cardiovascular and respiratory conditions were stable, mechanical ventilation was interrupted and the patient was returned to high-flow oxygen supplementation. On August 21, blood O_2_ saturation was 96% and supplemental oxygen was reduced to low-flow. Patient presented no fever and an additional dose of COVID-19 convalescent donor plasma (200 mL, 1/320 nAb) was administered. On August 22, patient was stable, with no fever and a new lung-CT scan revealed remarkable improvement scoring 11 (Fig. 1e). On August 24, a final dose of COVID-19 convalescent donor plasma (200 mL, 1/320 nAb) was administered; patient was removed from supplemental oxygen and blood O_2_ saturation was 97%. On August 26, patient was discharged presenting blood O_2_ saturation 94% on ambient air. Anti-SARS-CoV-2 IgM antibodies scored positive, whereas IgG scored borderline. As for September 21, patient was asymptomatic and free from SARS-CoV-2 infection.

## Discussion and conclusions

This is a single patient report that adds to other few reports on CVID and agammaglobulinemia, suggesting that COVID-19 convalescent donor plasma could be a valuable therapeutic approach to treat patients affected by dysgammaglobulinemias and presenting severe COVID-19 [6–10, 14]. Further studies, with larger cohorts should be performed to determine the detailed therapeutic outcomes of COVID-19 convalescent donor plasma in patients with abnormal capacity to produce antibody responses.

## Data Availability

Does not apply.

## Abbreviations

COVID-19: Coronavirus diseases-2019
CT: Computed tomography
CVID: Common variable immunodeficiency
ICON: International Consensus Document: Common Variable Immunodeficiency Disorders
nAB: Neutralizing antibodies
